# Removal of H2A.Z by INO80 promotes homologous recombination

**DOI:** 10.15252/embr.201540330

**Published:** 2015-07-03

**Authors:** Hanan E Alatwi, Jessica A Downs

**Affiliations:** MRC Genome Damage and Stability Centre, University of SussexFalmer, Brighton, UK

**Keywords:** ANP32E, chromatin, H2A.Z, homologous recombination, INO80

## Abstract

The mammalian INO80 remodelling complex facilitates homologous recombination (HR), but the mechanism by which it does this is unclear. Budding yeast INO80 can remove H2A.Z/H2B dimers from chromatin and replace them with H2A/H2B dimers. H2A.Z is actively incorporated at sites of damage in mammalian cells, raising the possibility that H2A.Z may need to be subsequently removed for resolution of repair. Here, we show that H2A.Z in human cells is indeed rapidly removed from chromatin flanking DNA damage by INO80. We also report that the histone chaperone ANP32E, which is implicated in removing H2AZ from chromatin, similarly promotes HR and appears to work on the same pathway as INO80 in these assays. Importantly, we demonstrate that the HR defect in cells depleted of INO80 or ANP32E can be rescued by H2A.Z co-depletion, suggesting that H2A.Z removal from chromatin is the primary function of INO80 and ANP32E in promoting homologous recombination.

## Introduction

The INO80 family of chromatin remodelling enzymes is defined by an insertion in the ATPase domain and, in addition to INO80, includes yeast Swr1 and human p400 and SRCAP. Evidence suggests that members of this family are capable of histone exchange reactions (for review, see 1). Swr1, as part of the SWR complex, removes H2A/H2B dimers and replaces them with H2A.Z/H2B 2,3. Mammalian SRCAP and p400 (as part of TIP60) are related to yeast SWR and perform the same function 4,5. The budding yeast INO80 complex has been shown to catalyse the reverse reaction and replace H2A.Z/H2B dimers with H2A/H2B 6. In mammalian cells, the histone chaperone ANP32E has been shown to remove H2A.Z from chromatin 7,8, but it has not been investigated whether mammalian INO80 contributes to this activity.

INO80 has been widely implicated in homologous recombination 9-11,12-14,15-17,18-20. There is evidence in both yeast and mammalian cells that INO80 functions to promote resection 9-18,19, and although the defect in mammalian cells depleted of INO80 is relatively mild, this may be sufficient to impair efficient HR.

Recently, incorporation of H2A.Z by TIP60 at damaged chromatin was found to restrict resection 5. In the absence of H2A.Z, recruitment of the non-homologous end-joining (NHEJ) complex Ku70/Ku80 to DNA breaks is impaired, and this appeared to be a consequence of unrestricted resection 5. Taken together, these data raise the possibility that INO80 may promote HR by removing H2A.Z to allow resection. We investigated this possibility and found that H2A.Z is incorporated and then very rapidly removed from chromatin following DNA damage. We found that the removal of H2A.Z from chromatin is dependent on INO80. Notably, while the depletion of H2A.Z does lead to increased RPA foci, consistent with a role in preventing unrestricted resection, we find that the depletion of INO80 has only marginal effects on resection. Instead, we find that cells are unable to efficiently replace RPA with RAD51, and consequently, the formation of sister chromatid exchanges is impaired in the absence of INO80. These data suggest that H2A.Z removal performs an additional function during HR that is separated from regulating resection. We also investigated the potential role of ANP32E in mediating HR and found that its depletion results in a similar defect as loss of INO80, and they appear to function together in mediating HR. Strikingly, we find that the co-depletion of H2A.Z and either INO80 or ANP32E fully rescues the defects in RAD51 foci and SCE formation of siINO80 or siANP32E cells, suggesting that the primary function of INO80 and ANP32E in promoting HR is the removal of H2A.Z from damaged chromatin.

## Results and Discussion

### H2A.Z dynamics at damaged chromatin

**Figure 1:**
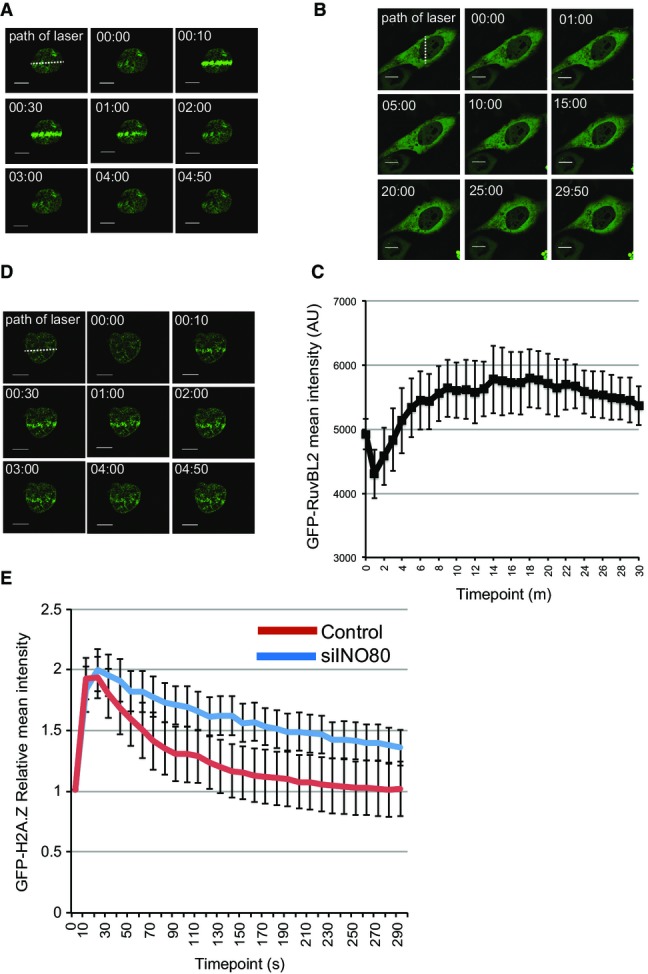
H2A.Z dynamics at damaged chromatin H2A.Z is rapidly incorporated and removed from chromatin in the vicinity of damaged DNA. U2OS cells transfected with GFP-H2A.Z were laser micro-irradiated and monitored by live cell imaging. Representative images taken at indicated time points are shown.

To investigate the possibility that H2A.Z is removed from chromatin subsequent to its incorporation after DNA damage, we introduced a GFP-tagged expression construct into cells. Using live cell imaging, we monitored H2A.Z accumulation at DNA damage sites induced by laser micro-irradiation. Consistent with previous findings 5, we found that H2A.Z is incorporated after damage (Fig1A). Strikingly, we found that it is subsequently very rapidly removed from chromatin, with signal intensity returning to pre-damage levels within 3 min (Fig1A and E). This is distinct from the behaviour of GFP-tagged core histone H2B, which we find shows no detectable patterns of movement under these conditions (Fig EV1A). This result suggests that H2A.Z is actively removed from damaged chromatin after it is incorporated.

**Figure:**
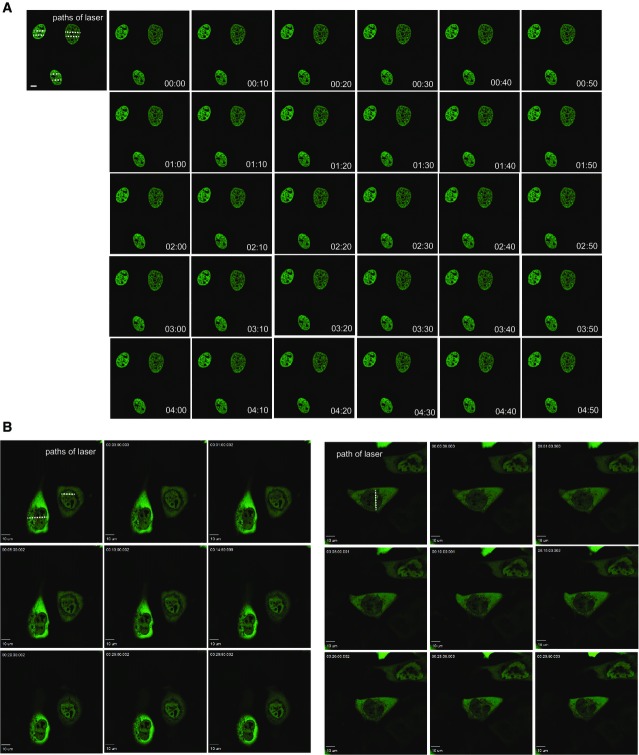
Histone dynamics at damaged chromatin A, B U2OS cells transfected with GFP-tagged H2B (A) or EGFP-tagged RuvBL2 (B) were laser micro-irradiated and monitored by live cell imaging. Representative images taken at indicated time points are shown. Data information: Scale bars represent 10 μm.

The RuvBL2 subunit of INO80 accumulates at sites of damaged DNA. U2OS cells transfected with EGFP-RuvBL2 were laser micro-irradiated and monitored by live cell imaging. Representative images taken at indicated time points are shown.

Quantification of mean fluorescence intensity at sites of micro-irradiation ± SE (*n* = 3).

Removal of H2A.Z from chromatin in the vicinity of damaged DNA is at least partly dependent on INO80. USOS cells treated with siINO80 were transfected with GFP-H2A.Z, laser micro-irradiated and monitored by live cell imaging. Representative images taken at indicated time points are shown.

Quantification of relative mean fluorescence intensity ± SD at sites of micro-irradiation in control (U2OS; *n* = 3) and siINO80 cells (*n* = 3) in a minimum of 10 cells per experiment. Data information: Scale bars represent 10 μm.

While budding yeast INO80 is capable of removing H2A.Z from chromatin and replacing it with H2A 6, this has not been investigated for mammalian INO80. INO80 has previously been shown to accumulate at DNA DSBs when analysed using chromatin immunoprecipitation 15. We analysed the dynamics of the RuvBL2 subunit of INO80, and in agreement with the ChIP data 15, we find that it accumulates in chromatin in proximity to damage induced by laser micro-irradiation, but then remains in the vicinity of damaged chromatin until at least 15 min after damage (Figs1B, C and EV1B).

To determine whether H2A.Z removal following damage is dependent on INO80, we analysed GFP-H2A.Z dynamics after depleting INO80 (siINO80). We found that the accumulation of H2A.Z in laser-irradiated chromatin is unaffected by depletion of INO80. In contrast, the removal of accumulated H2A.Z in damaged chromatin is significantly slower in cells depleted of INO80 when compared with control cells (Fig1D and E), indicating that INO80 contributes to the removal of H2A.Z from chromatin in mammalian cells.

### INO80 facilitates multiple steps during HR

**Figure 2:**
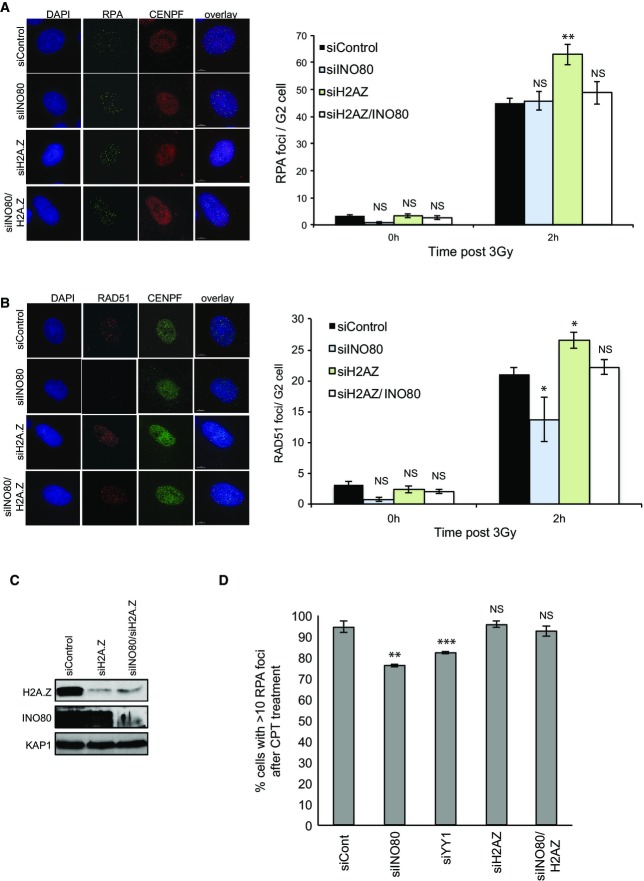
Cells lacking INO80 have a modest resection defect and a more significant defect in RAD51 foci accumulation, and these defects are rescued by depletion of H2A.Z IR induced RPA focus formation in A549 cells treated with siControl, siINO80, siH2A.Z or siINO80/siH2A.Z. Left hand panel: representative images. Right hand panel: quantification of foci.

There is evidence that INO80 contributes to HR in mammalian cells. In order to investigate the contribution of INO80 and H2A.Z to this pathway, we restricted our analyses to G2 cells using CENP-F, whose expression is limited to late S and G2 phases of the cell cycle 21, as a marker. There is evidence that the resection step of HR is defective in mammalian cells lacking INO80 15,18. We therefore monitored the appearance of RPA foci following irradiation as a readout of single-stranded DNA formation. We did not detect a difference in the number of RPA foci in cells treated with siINO80 when compared with control cells (Fig2A). In contrast, and consistent with a previous report 22, we find that the depletion of SRCAP has an obvious impact on the number of IR-induced RPA foci (Fig EV2). The absence of an obvious defect in RPA foci formation is consistent with our previous work investigating the YY1 and RuvBL2 subunits of INO80 17, but is apparently at odds with other reports 15,18. We considered that one possibility for the discrepancy between these findings is the different methodologies and conditions used to monitor resection. We therefore used a different approach and monitored the number of cells with RPA foci following treatment with camptothecin (CPT), and, in doing so, found a modest but statistically significant defect in cells treated with siRNA targeting either the INO80 or YY1 subunits of the INO80 complex (Fig2D), suggesting that INO80 does function to promote resection following DNA double strand breaks.

**Figure:**
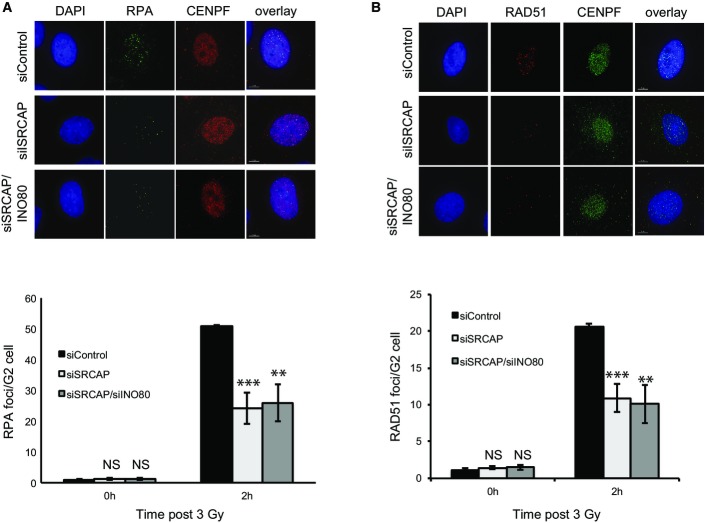
Cells lacking SRCAP have a resection defect IR induced RPA focus formation in A549 cells treated with siControl, siSRCAP or siSRCAP/siINO80. Upper panel: representative images. Lower panel: quantification of foci. When compared with control cells at 2 h, siSRCAP and siSRCAP/siINO80 are significantly different (***P = 0.0009 and **P =* *0.002, respectively).

IR induced RAD51 focus formation in cells as in (A). Upper panel: representative images. Lower panel: quantification of foci as in (A). When compared with control cells at 2 h, siSRCAP and siSRCAP/siINO80 are significantly different (***P = 0.00098 and **P =* *0.00248, respectively). Data information: Scale bars represent 5 μm. Data represent the mean of 3 independent assays ± SD. NS, not significant by Student’s *t*-test.

IR induced RAD51 focus formation in A549 cells treated with siControl, siINO80, siH2A.Z or siINO80/siH2A.Z. Left hand panel: representative images. Right hand panel: quantification of foci.

Western blot analysis showing efficiency of siRNA depletion. KAP1 is used as a loading control.

Quanitification of RPA foci following 1 h of treatment with camptothecin (CPT). Data information: Scale bars represent 5 μm. Data represent the mean of 4 (A, B) or 3 (D) independent assays ± SD. NS, not significant; *P ≤ 0.05; **P ≤ 0.01; ***P ≤ 0.001 by Student’s *t*-test.

We next monitored the accumulation of RAD51 foci following irradiation and found a more substantial defect in INO80-depleted cells (Fig2B). It seems unlikely that this is entirely a consequence of the minor resection defect in siINO80 cells, and it therefore suggests that INO80 has an additional function in promoting RAD51 foci formation during HR.

### Depletion of H2A.Z rescues the RAD51 foci formation defect of cells depleted of ANP32E and INO80

We hypothesised that the removal of H2A.Z from damaged chromatin by INO80 may be an important aspect of the ability of INO80 to promote RAD51 foci formation. To test this, we co-depleted H2A.Z and INO80 (Fig2C) and analysed the accumulation of RPA and RAD51 foci following DNA damage. We found that, while the depletion of H2A.Z alone had no detectable effect, the depletion of H2A.Z rescued the defect in RAD51 foci accumulation in siINO80 cells (Fig2B). Using the assay in which we can uncover defect in RPA foci formation in siINO80 cells, we find that the depletion of H2A.Z also rescues this defect (Fig2D). Loss of H2A.Z alone results in a greater number of RPA foci following irradiation (Fig2A), consistent with a previous report showing that H2A.Z is a barrier to resection 5. Interestingly, the number of RPA foci appears to be reduced when INO80 is also depleted back to wild-type levels (Fig2A), suggesting there may be crosstalk between the resection machinery and INO80.

**Figure 3:**
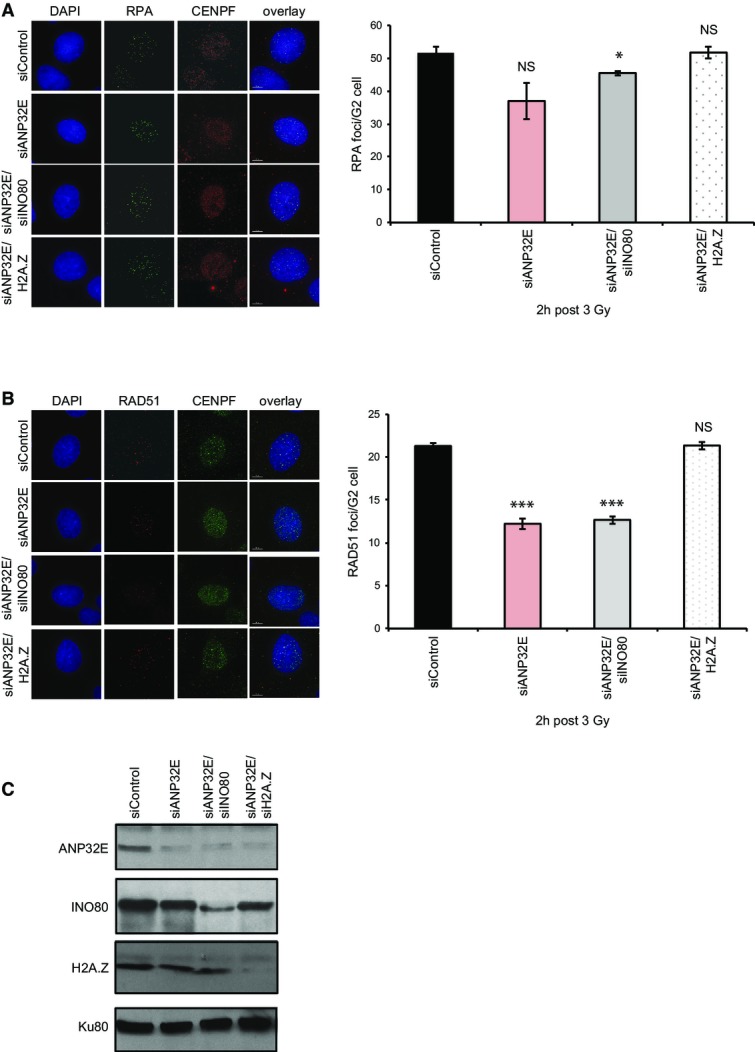
INO80 and ANP32E work together to promote RAD51 foci formation following irradiation, which is rescued by the depletion of H2A.Z IR induced RPA focus formation in A549 cells treated with siControl, siANP32E, siANP32E/siINO80 or siANP32E/siH2A.Z. Left hand panel: representative images. Right hand panel: mean number of foci ± SD.

Very recently, the histone chaperone ANP32E was shown to remove H2A.Z from chromatin in mammalian cells, both globally 7,8 and from chromatin at sites of damage 23. We were therefore interested in understanding whether ANP32E might contribute to HR in a similar manner to INO80. We depleted ANP32E and monitored RPA and RAD51 foci formation as previously, and we found that loss of ANP32E results in a similar defect in RAD51 foci formation following irradiation as we find with the depletion of INO80 (Fig3B). Notably, the depletion of both ANP32E and INO80 results in no further defects in RAD51 foci accumulation following irradiation (Fig3A–C), suggesting that they are working together to facilitate this step in HR. Most importantly, the co-depletion of H2A.Z in cells lacking ANP32E rescues the defects in foci formation of the siANP32E cells (Fig3B and C).

IR induced RAD51 focus formation in A549 cells treated with siControl, siANP32E, siANP32E/siINO80 or siANP32E/siH2A.Z. Left hand panel: representative images. Right hand panel: mean number of foci ± SD.

Western blot analysis showing efficiency of siRNA depletion. Ku80 is used as a loading control. Data information: Scale bars represent 5 μm. All data (A, B) are from a minimum of 3 independent assays. NS, not significant; *P ≤ 0.05; ***P ≤ 0.001 by Student’s *t*-test.

### Depletion of H2A.Z rescues the sister chromatid exchange (SCE) defect of cells depleted of ANP32E and INO80

The reduction in RAD51 foci formation in cells lacking INO80 or ANP32E suggests that HR is impaired in these cells. However, the reduction is modest when compared with cells lacking core HR proteins, such as BRCA2. We therefore monitored sister chromatid exchanges (SCEs) as a measure of HR completion. To do this, we treated cells with mitomycin C (MMC) because this results in a very substantial increase in SCEs, giving us the best possibility of detecting defects in the pathway. Additionally, unlike IR, MMC-induced lesions are repaired predominantly by HR.

**Figure 4:**
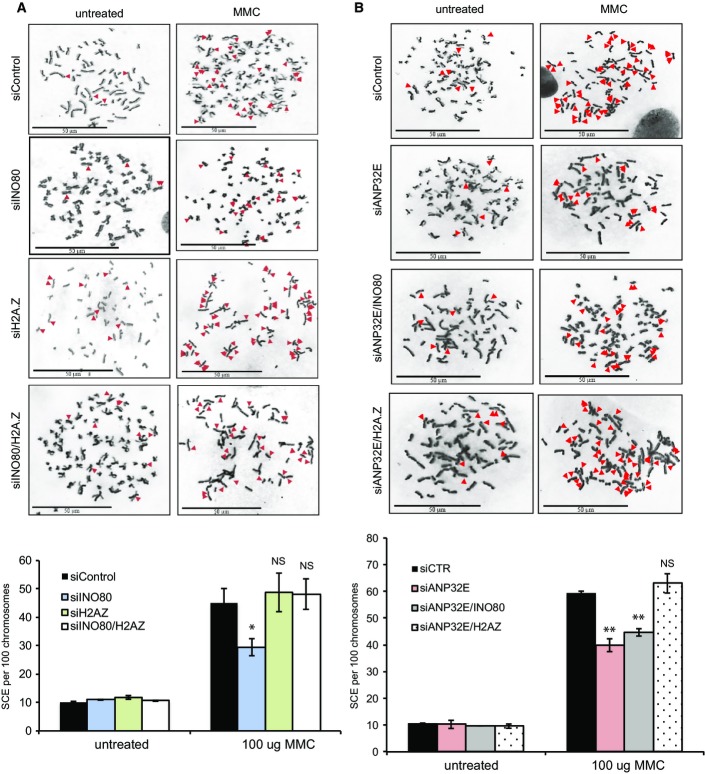
Wild-type levels of sister chromatid exchanges (SCEs) are dependent on H2A.Z removal by INO80 and ANP32E SCEs were monitored in HeLa cells treated with siControl, siINO80, siH2A.Z or siINO80/siH2A.Z following treatment with mitomycin C (MMC). The reduction in SCEs in cells lacking INO80 is rescued by the co-depletion of H2A.Z. Upper panel: representative images. Lower panel: mean number of SCEs ± SD. SCEs were scored in at least 1,000 chromosomes from three independent experiments.

We found that cells lacking either INO80 or ANP32E show reduced numbers of SCEs when compared with control cells (Fig4A and B). Depletion of H2A.Z alone had no detectable effect on SCE numbers after MMC treatment (Fig4A). Similar to our results monitoring RAD51 foci formation, we found that the co-depletion of H2A.Z rescued the defects in SCEs in cells lacking either INO80 or ANP32E (Fig4A and B). In addition, the depletion of both INO80 and ANP32E yielded similar levels of SCEs to cells lacking either INO80 or ANP32E (Fig4B), suggesting, as above, that they are working together to facilitate HR.

SCEs were monitored in HeLa cells treated with siControl, siANP32E, siANP32E/siINO80 or siANP32E/siH2A.Z. There is no further reduction in SCEs in cells depleted of both ANP32E and INO80 when compared with ANP32E alone. As with siINO80, the reduction in SCEs in cells lacking ANP32E can be rescued by the co-depletion of H2A.Z. Upper panel: representative images. Lower panel: mean number of foci ± SD. Data information: Scale bars represent 50 μm. All data are from a minimum of 3 independent assays. NS, not significant; *P ≤ 0.05; **P ≤ 0.01 by Student’s *t*-test.

Collectively our data suggest that H2A.Z is removed from chromatin after DNA damage by ANP32E and INO80, and this removal—either from the chromatin flanking the DSB, or from the sister chromatid, or both—is required for HR. Very recently, Price and colleagues also found that H2A.Z is removed from chromatin at sites of DNA damage in an ANP32E-dependent manner 23. Interestingly, they found that failure to remove H2A.Z from chromatin in the vicinity of damage results in reduced Ku70/Ku80 binding, which impairs NHEJ activity. Loss of H2A.Z leads to similar impairment of Ku70/Ku80 binding and NHEJ activity 5 and is consistent with a model in which the coupled incorporation and removal of H2A.Z is required to prevent promiscuous resection and promote NHEJ. While INO80 has been implicated in mediating resection, our data suggest that this is a minor role and that it has a greater impact on facilitating HR at a step downstream of resection. Taken together with the data from the Price laboratory, we speculate that H2A.Z removal has multiple roles in DSB responses. As we were restricting our analyses to late S and G2 cells, one intriguing possibility is that H2A.Z dynamics are used differentially throughout the cell cycle to promote distinct steps in both major DSB repair pathways. Importantly, if H2A.Z is not deposited into chromatin in the vicinity of DNA damage in the first place, then INO80 and ANP32E are no longer required for wild-type levels of RAD51 foci or SCE formation in these assays, indicating that the removal of H2A.Z from chromatin in the vicinity of DNA damage is their primary function in HR.

## Materials and Methods

### Cell culture and irradiation

A549, HeLa or U2OS cells were cultured in MEM or DMEM (Gibco), respectively, supplemented with 10% FCS, L-glutamine, penicillin and streptomycin (Gibco) at 37°C in a humidified 95% air and 5% CO_2_ atmosphere. Cells were irradiated by exposure to a ^137^Cs source.

### Small interfering RNA (siRNA) knockdown conditions

siRNA-mediated knockdown was achieved using HiPerFect Transfection Reagent (Qiagen) following the manufacturer’s instructions. siRNA duplexes (Dharmacon SMARTpool) were transfected into 4 × 10^5^ of logarithmically growing cells per condition. Cells were harvested 24 h later, retransfected with siRNA and were then seeded and grown for 48 h. The siRNA oligonucleotide used for ANP32E was 5′-CGGCUUCCCAGCUUAAAUA-3′ (Dharmacon).

### Antibodies

The primary antibodies used were as follows: γH2AX (Upstate Technology; 05-636) at 1:800 for IF, RPA (Merck Millipore; LS-C38952) at 1:100 for IF, RAD51 (Santa Cruz Biotechnology; SC-8349) at 1:200 and CENP-F (Abcam; ab108483) for IF, INO80 (Abcam; ab118787, and Bethyl; A303-371A) at 1:2,000 for WB, H2A.Z (Cell Signaling Technology; 2718S) at 1:1,000 for WB, ANP32E (Sigma-Aldrich; SAB2100124) at 1:1,000 for WB, KAP1 (Abcam; ab22553) at 1:1,000 for WB and KU80 (Abcam; ab33242) at 1:1,000 for WB.

The secondary antibodies used were as follows: FITC (Sigma-Aldrich; F0257) at 1:100 for IF, Cy3 (Sigma-Aldrich; C2306) at 1:200 for IF, AlexaFluor 488 (Invitrogen; A21206) at 1:400 for IF, Goat Anti-Rabbit Immunoglobulin/HRP (Dako; P0449) at 1:2,000 for WB, Rabbit Anti-Mouse Immunoglobulins/HRP (Dako; P044801-2) at 1:2,000 for WB.

### Immunofluorescence

A549 cells plated on glass slides were fixed for 10 min with fixative (2% (w/v) PFA, 3% (w/v) sucrose, 1× PBS) and permeabilised for 3 min with 0.2% Triton X-100 in PBS. When staining for RPA/RAD51, pre-extraction was performed by treatment with 0.2% Triton X-100 in PBS for 0.5–1 min prior to PFA fixation. Cells were rinsed with PBS and incubated with primary antibody diluted in PBS + 2% (w/v) BSA for 1 h at room temperature (RT). Cells were washed three times, incubated with secondary antibody (diluted in PBS + 2% (w/v) BSA) for 30 min at RT in the dark, incubated with 4′,6-diamidino-2-phenylindole (DAPI) for 10 min and washed three times with PBS. Slides were mounted using Vectashield and visualised/analysed using a Nikon-e400 microscope and imaged using an Applied Precision® Delta Vision® RT Olympus IX70 deconvolution microscope and softWoRx® Suite software. For γH2AX, RPA, and RAD51 foci quantification, a minimum of 30 cells per experiment was scored blindly and error bars represent the SD between three experiments.

For analysis in Fig2D, cells were treated for 1 h with 1 mM camptothecin (CPT; Sigma-Aldrich) and left to recover for 1 h. Recovered cells were then stained, and γH2AX-positive cells were scored as above in a minimum of 100 cells per experiment.

### Sister chromatid exchange (SCE) assay

Analysis of SCEs was carried out as described previously 24. Briefly, HeLa cells were grown for 48 h in BrdU and treated with 100 μg mitomycin C (MMC) for 16 h; then, 10 μg/ml colcemid was added for 2 h to collect cells in mitosis. SCEs were scored in at least 1,000 chromosomes from three independent experiments.

### Laser micro-irradiation

Exponentially growing human U2OS cells were plated onto 35-mm glass-bottom dishes (MatTek) and transfected with the pTGFP-H2AZ construct, pEGFP-RUVBL2 (Origene) or pEGFP-H2B using NanoJuice according to the manufacturer’s protocol. The cells were allowed to express the construct for 24 h and were then incubated with 10 μg/ml Hoechst 3458 for 30 min at 37°C before irradiation. The microscope system used was an Intelligent Imaging Innovations spinning disc confocal with a Yokogawa CSU-X1 on an Olympus IX-71. GFP-positive cells were irradiated with 405-nm ultraviolet laser set at power of 7:1,000 for either H2AZ or H2B and at 30:1,000 for RUVBL2 and channelled through a 60× objective. Images were captured at 10-s intervals following laser damage for a total time of 5 min for H2AZ or H2B and 30 min for RUVBL2. Images generated were acquired on a Photometrics Evolve 512 × 512 EMCCD using Slidebook 6 software. In protein recruitment experiments, signal intensity was quantified along the laser path, using Slidebook 6 software, in a minimum of 10 cells and error bars represent the SD between three independent experiments.

## Supporting Information

**Expanded View Figures PDF**

**Review Process File**
