# Supplementary material for: Removal of H2A.Z by INO80 promotes homologous recombination
Source: EMBO Rep. 2015 Jul 3;16(8):986–94. doi: 10.15252/embr.201540330 (PMC4552491; doi:10.15252/embr.201540330)
Supplement: Supplementary file 1 [file embr0016-0986-sd1.pdf]

Expanded View Figures

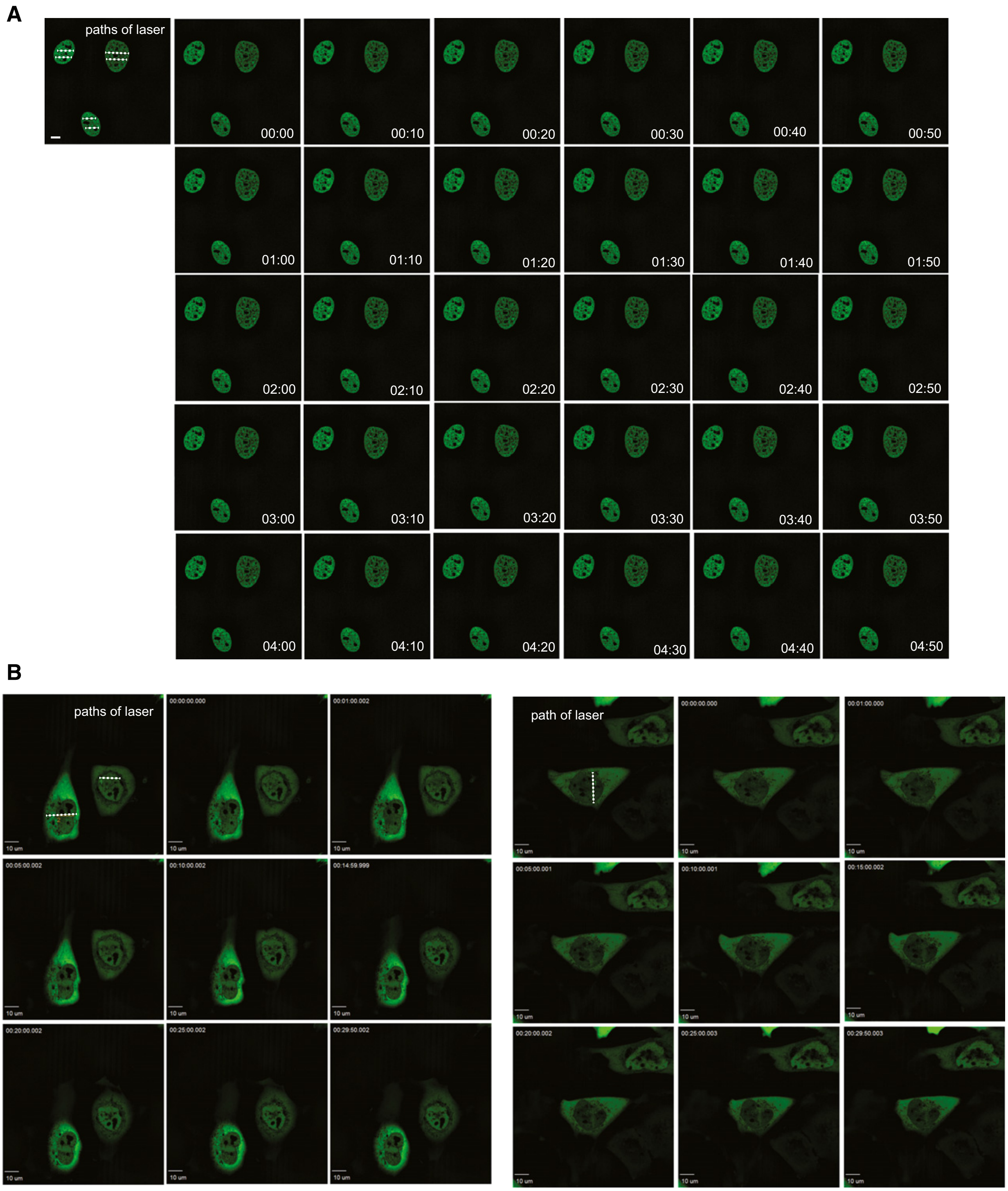

Figure EV1. Histone dynamics at damaged chromatin.

A, B U2OS cells transfected with GFP-tagged H2B (A) or EGFP-tagged RuvBL2 (B) were laser micro-irradiated and monitored by live cell imaging. Representative images taken at indicated time points are shown.

Data information: Scale bars represent 10 μm.

 © 2015 The Authors

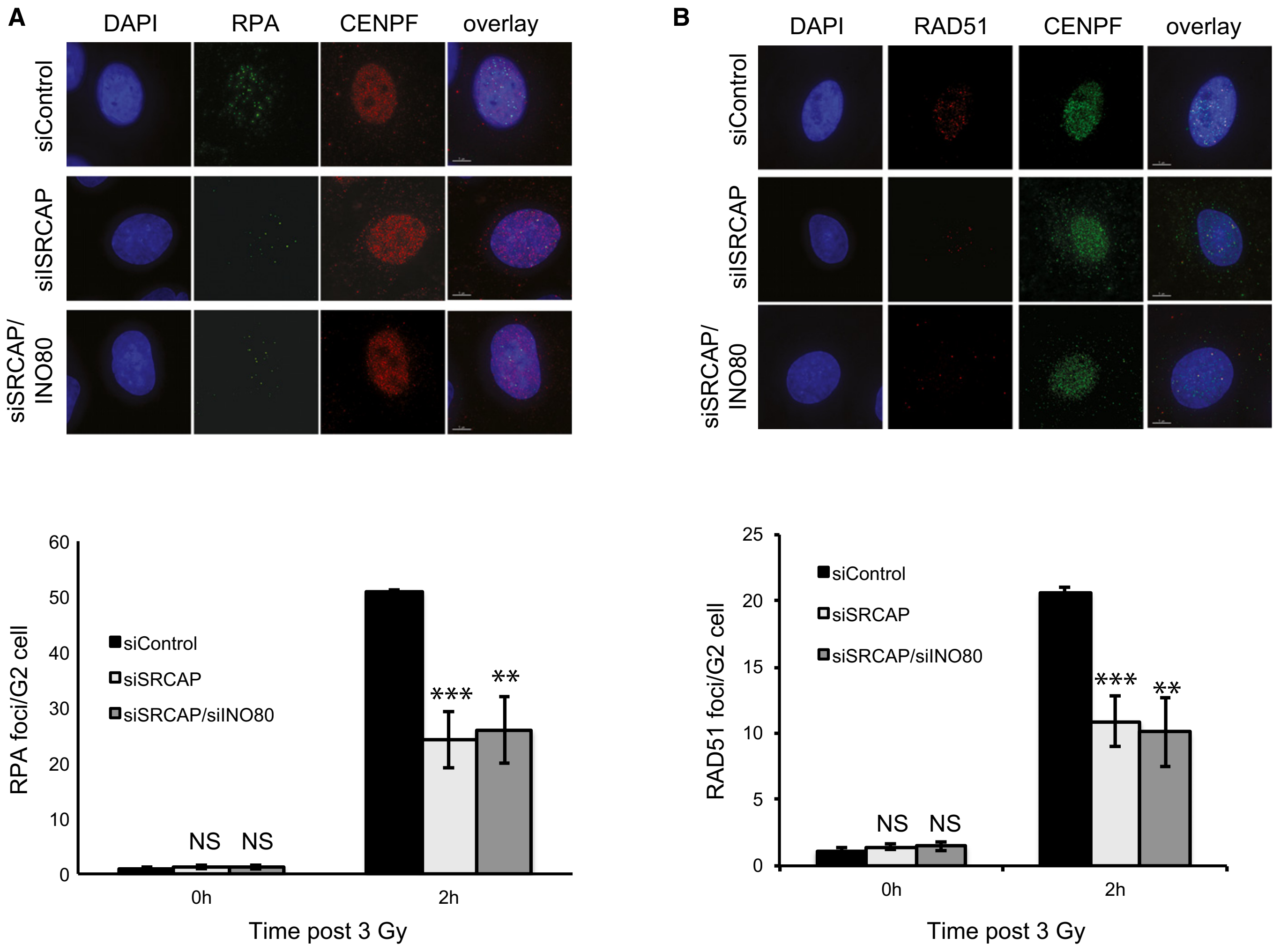

Figure EV2. Cells lacking SRCAP have a resection defect.

A IR induced RPA focus formation in A549 cells treated with siControl, siSRCAP or siSRCAP/siINO80. Upper panel: representative images. Lower panel: quantification of foci. When compared with control cells at 2 h, siSRCAP and siSRCAP/siINO80 are significantly different (***P = 0.0009 and **P = 0.002, respectively).

B IR induced RAD51 focus formation in cells as in (A). Upper panel: representative images. Lower panel: quantification of foci as in (A). When compared with control cells at 2 h, siSRCAP and siSRCAP/siINO80 are significantly different (***P = 0.00098 and **P = 0.00248, respectively).

Data information: Scale bars represent 5 μm. Data represent the mean of 3 independent assays ± SD. NS, not significant by Student's t-test.

© 2015 The Authors
