# Supplementary material for: Removal of H2A.Z by INO80 promotes homologous recombination
Source: EMBO Rep. 2015 Jul 3;16(8):986–94. doi: 10.15252/embr.201540330 (PMC4552491; doi:10.15252/embr.201540330)
Supplement: Supplementary file 2 [file embr0016-0986-sd2.pdf]

EMBO
reports

Manuscript EMBOR-2015-40330

Removal of H2A.Z by INO80 promotes homologous recombination

Hanan E. Alatwi and Jessica A. Downs

Corresponding author: Jessica A. Downs, University of Sussex

Review timeline:

Submission date:	05 March 2015
Editorial Decision:	23 March 2015
Revision received:	29 May 2015
Editorial Decision:	02 June 2015
Revision received:	09 June 2015
Accepted:	10 June 2015

Transaction Report:

(Note: With the exception of the correction of typographical or spelling errors that could be a source of ambiguity, letters and reports are not edited. The original formatting of letters and referee reports may not be reflected in this compilation.)

Editor: Esther Schnapp

1st Editorial Decision 23 March 2015

Thank you for the submission of your research manuscript to EMBO reports. We have now received the enclosed reports on it.

As you will see, the referees acknowledge that the findings are potentially interesting. However, they also have several technical concerns, including missing controls and statistical analyses, and all point out that the fluorescent images must be improved. Referee 3 adds that the role of NAP32E in H2A.Z eviction should be confirmed.

Given these constructive comments, we would like to invite you to revise your manuscript with the understanding that the referee concerns must be fully addressed and their suggestions taken on board. Acceptance of the manuscript will depend on a positive outcome of a second round of review. It is EMBO reports policy to allow a single round of revision only and acceptance or rejection of the manuscript will therefore depend on the completeness of your responses included in the next, final version of the manuscript.

Regarding data quantification, can you please specify the number "n" for how many experiments were performed, the bars and error bars (e.g. SEM, SD) and the test used to calculate p-values in the respective figure legends? This information is currently incomplete and must be provided in the figure legends. Please also include scale bars in all microscopy images.

© European Molecular Biology Organization

I look forward to seeing a revised version of your manuscript when it is ready. Please let me know if you have questions or comments regarding the revision.

REFEREE REPORTS:

Referee #1:

The INO80 remodeling enzyme is important for optimal repair of DNA double strand breaks in both yeast and mammals. Most studies implicate INO80 in the homologous recombination pathway of repair, but the precise role of INO80 is not clear. Work in yeast are a bit contradictory, with some studies implicating INO80 in the resection process, whereas other studies reporting no defect in this HR step. INO80 can remove the H2A.Z histone variant from chromatin, and work in yeast suggested that this activity may regulate the checkpoint response to DSBs. Little is known about the role of INO80 in HR repair in mammals. Here, Alatwi and Downs investigate the function of human INO80 in the DSB response. First, they use a H2A.Z-GFP fusion protein to follow its recruitment and retention to a stripe of laser irradiation. The data show that H2A.Z is rapidly recruited and rapidly removed. Interesting, knockdown of INO80 slows the removal of H2A.Z, consistent with its known biochemical activity. A similar effect is also observed when the Anp32E chaperone is removed. Furthermore, knockdown of INO80 causes a diminished formation of Rad51 foci, with little effect on RPA foci. Loss of INO80 also leads to fewer sister chromatid exchanges (SSEs) in response to mitomycin.

This work nicely links the H2A.Z removal activity of INO80 to mammalian DSB repair. Although the data are generally solid, there are a few technical deficiencies. First, the RPA and Rad51 foci images in Figures 2 and 3 are extremely weak in intensity, precluding evaluation. The Rad51 foci are not visible at all to this reviewer. Better images need to be provided. Second, the authors need to provide western blot images that document the knockdown efficiency of Anp32E. This is especially important for the double knockdown with INO80. Westerns for INO80 should also be included here.

Referee #2:

This is an excellent albeit brief demonstration that the HS Ino80 complex removes H2A.z at damage. It is suitable for a brief communication. Funtional readout is limited, but relevance for the field is very high. Some care could be given to make the fluorescence images clearer . Controls with a SRCAP -Ino80 double knockdown would be desirable, if technically feasible.

Referee #3:

Removal of H2A.Z by INO80 promotes homologous recombination

Alawi and Downs

In this manuscript, the authors described the rapid incorporation and the following rapid eviction of the histone variant H2A.Z at DNA damage sites. They then proposed that the INO80 chromatin remodeling complex has a role in homologous recombination repair through the eviction of H2A.Z from the DNA damage sites. In addition to INO80 complex, the histone chaperon ANP32E was supposed to be involved in DNA repair through the H2A.Z-eviction together with the INO80 complex. These results contains novel aspects of chromatin structure involved in DNA repair, and would give an explanation of previous observations about the involvement of INO80 complex and H2A.Z in DNA repair processes. However, some results are not enough convincing because of lack of control/supporting experiments and statistical analyses.

© European Molecular Biology Organization

Specific comments:
1. In Fig. 1A, any other canonical histone tagged with GFP should be analyzed as a control.
2. In Fig. 1B, GFP-RuvBL2 seems to be increased not only in the irradiated region, but also in other area of the nucleus. The author should repeat the experiment and show convincing images.
3. In Figs 2 and 3, the authors used CENPF as a control. However, centromeres are not clearly visualized by CENPF, and the authors did not mentioned about the CENPF images in the text. Author should explain the images of CENPF or should use any other antibody as an appropriate control.
4. In Figs 2A, 2B, 3A, 3B, 4A, and 4B, statistical comparison of the data was not performed (p-values were not given in the graphs).
5. In Fig. 3, western blotting of siNAP32E, siNAP32E/siINO80, and siNAP32E/H2A.Z cells should be shown.
6. In Figs 2A, 2B, 3A, and 3B, the IF images of RPA and RAD51 are not clearly visible. These images should be replaced.
7. H2A.Z-dynamics at DNA damage sites should be analyzed with siNAP32E cells to confirm the contribution of NAP32E to the eviction of H2A.Z.

1st Revision - authors' response 29 May 2015

Responses to Referees

We thank the referees for their positive comments and constructive suggestions. We've revised the manuscript accordingly, as described in detail below, and feel that the manuscript is much improved as a result of these changes.

Referee #1:

The INO80 remodeling enzyme is important for optimal repair of DNA double strand breaks in both yeast and mammals. Most studies implicate INO80 in the homologous recombination pathway of repair, but the precise role of INO80 is not clear. Work in yeast are a bit contradictory, with some studies implicating INO80 in the resection process, whereas other studies reporting no defect in this HR step. INO80 can remove the H2A.Z histone variant from chromatin, and work in yeast suggested that this activity may regulate the checkpoint response to DSBs. Little is known about the role of INO80 in HR repair in mammals. Here, Alatwi and Downs investigate the function of human INO80 in the DSB response. First, they use a H2A.Z-GFP fusion protein to follow its recruitment and retention to a stripe of laser irradiation. The data show that H2A.Z is rapidly recruited and rapidly removed. Interesting, knockdown of INO80 slows the removal of H2A.Z, consistent with its known biochemical activity. A similar effect is also observed when the Anp32E chaperone is removed. Furthermore, knockdown of INO80 causes a diminished formation of Rad51 foci, with little effect on RPA foci. Loss of INO80 also leads to fewer sister chromatid exchanges (SSEs) in response to mitomycin.

This work nicely links the H2A.Z removal activity of INO80 to mammalian DSB repair. Although the data are generally solid, there are a few technical deficiencies. First, the RPA and Rad51 foci images in Figures 2 and 3 are extremely weak in intensity, precluding evaluation. The Rad51 foci are not visible at all to this reviewer. Better images need to be provided.

We apologize for the difficulty with the images, which was picked up on by all the referees. We have collected new, higher resolution images and created new figures with these. We tested the quality of these images when opened using different operating systems and programs and when printed on several printers, and are satisfied that the foci are much more apparent.

Second, the authors need to provide western blot images that document the knockdown efficiency of Anp32E. This is especially important for the double knockdown with INO80. Westerns for INO80 should also be included here.

We are sorry for the omission and have included Western blot analyses in the revised manuscript.

Referee #2:

This is an excellent albeit brief demonstration that the HS Ino80 complex removes H2A.z at damage. It is suitable for a brief communication. Funtional readout is limited, but relevance for the field is very high. Some care could be given to make the fluorescence images clearer.

As described above, we have used new images to improve the clarity of these figures.

Controls with a SRCAP -Ino80 double knockdown would be desirable, if technically feasible.

This is a nice suggestion, and when we tested depletion of SRCAP (alone and in combination with INO80), we found a much more substantial reduction in RPA foci, consistent with this complex having a greater impact on resection than INO80. These data are included in revised Expanded View Figure S2.

Referee #3:

Removal of H2A.Z by INO80 promotes homologous recombination

Alawi and Downs

In this manuscript, the authors described the rapid incorporation and the following rapid eviction of the histone variant H2A.Z at DNA damage sites. They then proposed that the INO80 chromatin remodeling complex has a role in homologous recombination repair through the eviction of H2A.Z from the DNA damage sites. In addition to INO80 complex, the histone chaperon ANP32E was supposed to be involved in DNA repair through the H2A.Z-eviction together with the INO80 complex. These results contains novel aspects of chromatin structure involved in DNA repair, and would give an explanation of previous observations about the involvement of INO80 complex and H2A.Z in DNA repair processes. However, some results are not enough convincing because of lack of control/supporting experiments and statistical analyses.

Specific comments:
1. In Fig. 1A, any other canonical histone tagged with GFP should be analyzed as a control.

We have performed this control using GFP-tagged H2B and included it in revised Expanded View Figure S1A. We find that there is no detectable change in the pattern of H2B under these conditions.

2. In Fig. 1B, GFP-RuvBL2 seems to be increased not only in the irradiated region, but also in other area of the nucleus. The author should repeat the experiment and show convincing images.

We found it was difficult to get better images of the GFP-tagged RuvBL2 subunit, so we created an EGFP-tagged construct to test in these assays and this has improved the quality of the data we obtained (in revised Figure 1). Nevertheless, the enrichment of RuvBL2 at laser-microirradiated chromatin is slight, so we provided several additional examples in revised Expanded View Figure S1B to give an indication of reproducibility.

© European Molecular Biology Organization

3. In Figs 2 and 3, the authors used CENPF as a control. However, centromeres are not clearly visualized by CENPF, and the authors did not mentioned about the CENPF images in the text. Author should explain the images of CENPF or should use any other antibody as an appropriate control.

We apologize for the lack of explanation and have added text to the revised manuscript to clarify that CENPF staining was used to restrict our analyses to cells in G2.

4. In Figs 2A, 2B, 3A, 3B, 4A, and 4B, statistical comparison of the data was not performed (p-values were not given in the graphs).

We have performed paired t-tests to calculate p values and annotated the figures, figure legends and methods to include this information.

5. In Fig. 3, western blotting of siNAP32E, siNAP32E/siINO80, and siNAP32E/H2A.Z cells should be shown.

We are sorry for the omission and have included Western blot analyses in the revised manuscript.

6. In Figs 2A, 2B, 3A, and 3B, the IF images of RPA and RAD51 are not clearly visible. These images should be replaced.

As described above, we have used new images to improve the clarity of these figures.

7. H2A.Z-dynamics at DNA damage sites should be analyzed with siNAP32E cells to confirm the contribution of NAP32E to the eviction of H2A.Z.

Very recently, a manuscript from another group (Brendan Price and colleagues) has demonstrated that H2A.Z removal from chromatin flanking damaged DNA is dependent on ANP32E and is now in press at PNAS, so we have cited this in our revised manuscript.

2nd Editorial Decision 02 June 2015

Thank you for the submission of your revised manuscript to our journal. We have now heard back from referee 1 who was asked to assess it, and who supports publication of the manuscript now.

I only would like to ask you for a few minor changes before we can proceed with the official acceptance.
I noticed that the panels for figures 1C and 1E state n=2, in which case no statistics can be calculated. Can you please either repeat the experiment one more time, or remove the error bars and show the single data points of both experiments instead, along with their mean.

I am looking forward to receiving the final manuscript as soon as possible, so that we can go ahead and accept it.

REFEREE REPORTS:

Referee #1:

The authors have done an outstanding job at responding to reviewer comments. In particular, the new IF images are now very convincing. The newly provided western blot controls also alleviate my other concerns. This is a very nice piece of work that should be published in EMBO Reports.

2nd Revision - authors' response 09 June 2015

We have done one extra repeat of the laser tracking in Figure 1, and made all of the other minor changes as you suggested. Please do let me know if there is any additional information I can supply. Thanks again for all your help with our manuscript.

3rd Editorial Decision 10 June 2015

I am very pleased to accept your manuscript for publication in the next available issue of EMBO reports.

Thank you for your contribution to EMBO reports and congratulations on a successful publication. Please consider us again in the future for your most exciting work.

© European Molecular Biology Organization
